# Aggression among 216 patients with a first-psychotic episode of bipolar I disorder

**DOI:** 10.1186/s40345-018-0126-8

**Published:** 2018-08-11

**Authors:** Hari-Mandir K. Khalsa, Ross J. Baldessarini, Mauricio Tohen, Paola Salvatore

**Affiliations:** 1 International Consortium for Bipolar & Psychotic Disorders Research, Psychotic Disorders Division, McLean Hospital, Belmont, MA 02478 USA; 2 Division of Mental Health and Wellbeing, Warwick Medical School, Coventry, UK; 3 Department of Psychiatry, Harvard Medical School, Boston, MA 02215 USA; 4 Department of Psychiatry & Behavioral Sciences, University of New Mexico, Health Sciences Center, Albuquerque, NM 87131 USA; 5 Psychiatry Section, Department of Medicine & Surgery, University of Parma, Parma, Italy

**Keywords:** Aggression, Bipolar disorder, Psychosis, Violence

## Abstract

**Background:**

Aggression by patients with bipolar I disorder (BD-I) is not uncommon. Identifying potential risk factors early in the illness-course should inform clinical management and reduce risk.

**Methods:**

In a study sample of 216 initially hospitalized, first-psychotic episode subjects diagnosed with DSM-IV-TR BD-I, we identified recent (within 1 month before hospitalization) aggression by ratings on the Brief Psychiatric Rating Scale-Expanded and review of detailed clinical research records. We compared subjects with versus without aggressive behavior for associations with selected demographic and clinical factors.

**Results:**

Aggression was identified in 23/216 subjects (10.6%). It was associated significantly with recent suicide attempt (OR = 4.86), alcohol abuse (OR = 3.63), learning disability (OR = 3.14), and initial manic episode (OR = 2.59), but not with age, sex, onset-type, personality disorder, time to recovery, or functional status.

**Conclusions:**

Among first-major episode BD-I patients with psychotic features, recent serious aggression towards others was identified in 10.6%. The odds of aggression increased by 4.9-times in association with a recent suicide attempt, more than 3-times with alcohol-abuse or learning disability, and by 2.6-times if the episode polarity was manic. The findings encourage closer management of alcohol misuse, suicide risk, and manic symptoms, and early detection of learning problems in BD-I patients.

## Background

Rates for injuries or fatalities associated with aggression or violence (homicide, suicide, vehicular crashes) are reported to be 2 to 6-times higher among persons with a major mental illness than in the general population (CDC 2011). Identification of factors that predict such events may guide interventions aimed at limiting their risk. Few studies have addressed aggressive behaviors by persons diagnosed with untreated- or first-episode affective or non-affective psychotic disorders, and have considered schizophrenia more than bipolar disorder (Dean et al. 2007; Ballester et al. 2012; Volavka 2013; Winsper et al. 2013; Wan et al. 2014; Wasser et al. 2017). Accordingly, we evaluated the prevalence of aggressive behaviors and their clinical and demographic correlates by first major episode in patient-subjects at first-lifetime hospitalization with a stable research diagnosis of bipolar I disorder (BD-I) with psychotic features, at 2 years of follow-up, who were enrolled and followed in the McLean-Harvard First Episode Project.

## Methods

Study research methods were detailed previously (Tohen et al. 2003; Baethge et al. 2005; Salvatore et al. 2014). Briefly, experienced evaluators recruited subjects consecutively over 8 years (1989–1996) from inpatient units at McLean Hospital within 72 h of first-lifetime psychiatric hospitalizations. Subjects in the present study initially met DSM criteria for an episode of psychotic affective or non-affective illness; those in the present study had a stable diagnosis of BD-I at 2 years of follow-up. Subjects provided written informed consent for McLean Hospital IRB-approved study procedures. Exclusion criteria were: (a) current intoxication or substance-withdrawal; (b) delirium; (c) psychiatric hospitalization prior to the index admission, unless for detoxification-only; (d) dementia or other cognitive impairment, IQ < 70; (e) ill > 1 year prior to the index hospitalization; or (f) ever treated before intake with an antidepressant, mood-stabilizer or antipsychotic for a total of ≥ 3 months. Treatment was determined and supervised by fully independent treating clinicians, but was documented during initial hospitalization and at systematic follow-up assessments at 6, 12, and 24 months.

### Clinical assessment

Baseline information was collected by specifically trained Master’s-level research assistants with ≥ 5-years of experience; raters were repeatedly tested to maintain high inter-rater reliability (ICC ≥ 0.90). Demographic and salient clinical data, supplemented with symptom rating-scale scores, were collected from medical records, initial Structured Clinical Interviews for DSM-Patient Version (SCID-P) assessments, as well as semi-structured interviews of patients, relatives, and treating clinicians. Diagnoses, including co-occurring personality disorders and substance use disorders, were determined by a best-estimate procedure (Leckman et al. 1982; Tohen et al. 2003) at the index episode and reviewed comprehensively and verified as meeting DSM criteria at 2 years of follow-up, based on SCID assessments (baseline and 24-months) and all other available clinical information. Follow-up assessments were conducted by a rater blind to baseline information, as detailed previously (Tohen et al. 2003). Following the same best-estimate procedure, diagnoses that initially met criteria for DSM-III were updated to meet DSM-IV-TR criteria (APA 1995; Salvatore et al. 2007).

Assessment scales collected prospectively included a 36-item Expanded Brief Psychiatric Rating Scale (BPRS-E), including Item-10 “Hostility” (range 0–7: 0 = not assessed, 1 = not present, 2 = very mild, 3 = mild, 4 = moderate, 5 = moderately severe, 6 = severe, and 7 = extremely severe) (Tohen et al. 2003). BPRS-E “Hostility” was rated for the previous week and was based on self-report during patient interviews.

Systematic chart review (by PS) of admission summaries, rating scales, and all available research notes and clinical narratives determined the presence of physical aggression characterized as serious acts of violence or expressed intent to commit serious or lethal acts by the subject within 4 weeks of intake. Only the documented presence of serious physical aggression, including assault with homicidal ideation and behaviors and attempted homicide, during the first-lifetime major episode of psychotic BD-I was considered for the present analyses.

Presence of a learning disability was coded as present/absent, based on inpatient admission assessments, clinical notes and all available reports, including neuropsychological testing reports on neurocognitive function, that included any mention of impairment of “language”, “verbal” function, “auditory coordination”, or “speech”. Any learning disability that had emerged during antecedent or prodromal phases, including impairments in speaking, listening, writing, reading, spelling, reasoning, or doing mathematics, also was included.

### Statistical analyses

Potential covariates of aggressive behavior by subjects included: (a) illness-onset type, rated as *acute* (arising over < 1 month), *subacute* (1–6 months) or *gradual* (> 6 months); (b) BPRS-E total and (c) “Hostility” scores at baseline; (d) age at onset; (e) intake clinical presentation [manic vs. other (depressed, mixed, or non-affective psychosis)]; (f) presence of substance abuse within 6 months (alcohol, drug, or any) of index hospital admission; (g) documented learning disability; (h) any personality disorder; (i) suicide attempt within 6 months prior to index hospitalization; (j) syndromal remission; (k) relapse or recurrence; and (l) functional recovery.

Data are reported as mean ± standard deviation (SD) or with 95% confidence intervals (CI). Incidents of physical aggression were compared in subgroups-of-interest using likelihood-ratio tests (bivariable logistic regression). Median latencies to recovery and to new episodes (in weeks with 95% confidence intervals [CIs]) from the date of intake to clinical recovery, or to new episodes from the estimated start-date of recovery, were compared by Kaplan–Meier life-table survival analyses, with Mantel–Cox log-rank (χ^2^) tests. Variables with tentative bivariable associations (*p *≤ 0.10) were entered into multivariable logistic regression modeling to determine Odds Ratios (ORs) with CI. Analyses were performed with commercial microcomputer programs (Stata-12^®^, Stata Corp., College Station, TX; and Statview-5^®^, SAS Institute, Cary, NC for data spreadsheets).

## Results

### Intake demographic and clinical features

The study included 216 subjects; 117 (54.2%) were men, 173 (80.1%) were Caucasian, and onset-age averaged 31.2 (SD = 13.1) years. All subjects met DSM-IV-TR diagnostic criteria for BD-I with psychotic features over 2 years of systematic follow-up. Initial presentations included: bipolar I mania (n = 134; 35.9%), bipolar I mixed (73; 33.8%), major depression with psychotic features (5; 2.31%), or non-affective psychosis (4; 1.85%). Onset-types ranked: acute (131; 60.6%) > subacute (72; 33.3%) > gradual (13; 6.02%). Substance abuse was diagnosed in 143 (66.2%) subjects: 133 (61.6%) with alcohol abuse and 81 (37.5%) with drug abuse, within 6 months before intake. Co-occurring personality disorders were identified in 20 subjects (9.26%), and learning disability in 19 (8.80%). Suicide attempt within 1 month before intake occurred in 30 cases (13.9%).

### Aggressive acts and covariates

**Fig. 1:**
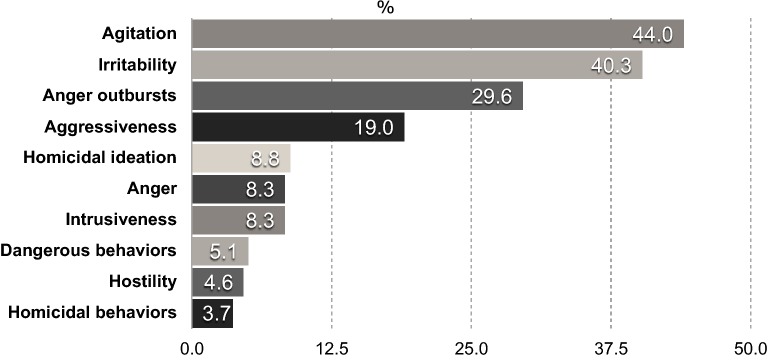
Aggressive symptoms and behaviors in 216 -BD-I patient-subjects. Serious aggressive acts seen in 23/216 (10.65%) were determined by demonstrated aggressiveness, homicidal behaviors, and dangerous behaviors towards others plus homicidal ideation. Percentages are greater than 100% due to patient-subjects exhibiting more than one act of aggression and related symptoms

Aggressive acts were identified in 23 (10.65%) of the 216 subjects, in combination with hostility, anger, homicidal ideation, and other clinically determined serious aggressiveness (Fig. 1). Ratings of hostility were in the mild to moderate range in 182/216 (84.3%) on the BPRS-E Item-10 (Hostility) scores, which averaged 2.46 ± 1.52 out of 7.00.

Several characteristics were significantly more prevalent among subjects with aggressive behavior (Table 1). These factors were entered into multivariable logistic regression modeling and ranked (by OR) as: (a) recent suicide attempt (OR = 4.86, *p *= 0.003), (b) alcohol abuse (OR = 3.63, *p *= 0.044), (c) learning disability (OR = 3.14, *p *= 0.049), and (d) initial presentation in mania (OR = 2.59, *p *= 0.002) (Table 2). There was no significant association of aggression with: sex, onset-age, rapidity of onset, diagnosis of personality disorder, or recent drug abuse. Patients with or without serious aggressive acts did not differ in time to remission or time to relapse, nor functional status.

**Table 1:** Factors associated with aggressive behaviors in 216 first-episode BD-I patient-subjects: bivariable logistic regression

Factor	Overall, N (%) or mean (SD)	Aggressive behaviors, N (%) or mean (SD)		Statistic^a^	*p*-value
		Present	Absent		
Subjects	216 (100.0)	23 (10.6)	193 (89.4)	–	–
Suicide attempt	30 (13.9)	7 (30.4)	23 (11.9)	4.81	*0.03*
Male	117 (54.2)	16 (69.6)	101 (52.3)	2.54	0.11
Onset age	31.2 (13.1)	35.0 (16.2)	30.8 (12.6)	1.95	0.16
Onset type					
Acute	131 (60.7)	13 (56.5)	118 (61.1)	0.18	0.67
Subacute	72 (33.3)	8 (34.8)	64 (33.2)	0.02	0.88
Gradual	13 (6.02)	2 (8.70)	11 (5.70)	0.29	0.59
Diagnosis at intake				5.81	*0.02*
Manic	134 (62.0)	15 (65.2)	119 (61.7)		
Mixed	73 (33.8)	5 (21.7)	68 (35.2)		
Depressed	5 (2.31)	1 (4.35)	4 (2.07)		
Psychosis NOS	2 (0.93)	1 (4.35)	1 (0.52)		
Brief psychosis	1 (0.46)	0 (0.00)	1 (0.52)		
Schizophreniform	1 (0.46)	1 (4.35)	0 (0.00)		
BPRS					
Total score	77.4 (23.0)	70.7 (21.2)	78.3 (23.2)	2.24	0.13
Hostility score	2.46 (1.52)	2.95 (1.66)	2.40 (1.50)	2.38	0.12
Substance abuse					
Any	143 (66.2)	19 (82.6)	124 (64.3)	3.42	0.06
Alcohol	133 (61.6)	19 (82.6)	114 (59.1)	5.33	*0.02*
Drug	81 (37.5)	9 (39.1)	72 (37.3)	0.03	0.86
Personality disorder^b^	20 (9.26)	2 (8.70)	18 (9.33)	0.01	0.92
Learning disability	19 (8.80)	5 (21.7)	14 (7.25)	4.14	*0.04*
Follow-up					
Remission (days)	93.2 (139.5)	74.2 (77.6)	95.3 (144.7)	0.47	0.49
Relapse (days)	513.3 (507.4)	542.3 (389.3)	510.3 (519.3)	0.04	0.85
Functioning (years/n)	58 (27.6)	7 (12.1)	51 (87.9)	0.28	0.59

Italic values indicate significance of *p*-value (*p* < 0.05)

^a^Results from bivariable logistic regression analysis with reported likelihood-ratio and *p*-value

^b^Personality disorders included cluster A (n = 3; paranoid, schizoid or schizotypal), cluster B (n = 15; antisocial, borderline histrionic, or narcissistic), and cluster C (n = 2; avoidant, dependent, obsessive–compulsive or other specified). The n = 2 with aggressive behaviors were diagnosed with cluster B personality disorders

**Table 2:** Multivariable logistic regression modeling: factors associated with aggressive behavior at first hospitalization in 216 first-episode BD-I subjects

Factor	OR (95% CI)	*z*-score	*p*-value
Suicidal acts	4.86 (1.69–13/9)	2.94	0.003
Alcohol abuse	3.63 (1.03–12.8)	2.01	0.044
Learning disability	3.14 (1.01–9.78)	1.97	0.049
Manic vs. other presentation	2.59 (1.41–4.78)	3.06	0.002

Model χ^2^ [*df* = 4] = 14.5, *p *= 0.006, N = 216. Other diagnoses at intake included: Bipolar I mixed episode (n = 73), major depressive episode (n = 5), and psychotic non-affective disorder [n = 4, including psychosis NOS (n = 2), schizophreniform disorder (n = 1), and brief psychotic disorder (n = 1)]. Factors are ranked by OR

## Discussion

This study was based on a sample of 216 newly diagnosed BD-I patients in a first-lifetime major illness episode with psychotic features, among whom 10.6% showed recent aggressive or violent behavior, very similar to the 11% found in a community survey by Swanson et al. (1990), cited in Feldman (2001). A particularly striking finding was a strong association with recent suicide attempt, suggesting common mechanisms contributing to aggression toward the self and toward others (Perroud et al. 2011; Tsiouris et al. 2011; Volavka 2013; Stefansson et al. 2015). Other significantly and independently associated factors included recent abuse of alcohol, a history of learning disability, and presentation in mania rather than mixed episodes, depressive episodes, or non-affective psychosis. Such findings may contribute to timely identification of persons at risk of violent behavior, including in the criminal justice system, as well as in clinical settings (Noga et al. 2014; Wan et al. 2014; Wasser et al. 2017), particularly with respect to first-episodes of bipolar or psychotic illness (Dean et al. 2007; Prince et al. 2007; Winsper et al. 2013; Wasser et al. 2017).

Previous findings have suggested an association between manic symptoms and aggressive behavior, whether in association with bipolar disorder or not (Feldmann 2001; Dean et al. 2007; Látalová 2009; Nielssen et al. 2012). Aside from primary psychiatric diagnosis, the present findings are consistent with previous reports of an association between substance abuse and aggressive and violent behavior (Amore et al. 2008; Colasanti et al. 2008; Látalová 2009; Ballester et al. 2012; Witt et al. 2013). Specifically, complex interactions of concomitant clinical factors and co-existing psychopathological features, including dual diagnosis of psychosis and addiction and co-occurrence of dimensions of impulsivity and impaired decision-making, both highly prevalent in psychotic as well as substance abuse disorders, perhaps more than the primary categorical diagnosis, may be predictive of violent behavior, including during hospitalization or acute phases of illness (Amore et al. 2008; Colasanti et al. 2008; Látalová 2009; Ballester et al. 2012; Witt et al. 2013). Although aggressive and non-aggressive BD-I patients were not distinguished by total BPRS scores, in a sample of 374 acutely ill, hospitalized subjects, higher levels of BPRS hostility-suspiciousness scores predicted more severe violent behavior, including physical rather than verbal (Amore et al. 2008). The high rate of suicide attempts found among the present BD-I subjects with aggressive behavior supports previous studies of associations between hostility or aggression and suicide attempts (Dean et al. 2007; Amore et al. 2008; Colasanti et al. 2008; Perroud et al. 2011; Tsiouris et al. 2011; Witt et al. 2013; Stefansson et al. 2015).

This study did not include subjects with intellectual disability (ID) based on IQ scores at least 2 standard deviations below the mean, or < 75, one of three diagnostic criteria for ID by the American Association of Intellectual and Developmental Disabilities; however, the presence of substantial risk (8.80%) of a learning disability in the present sample of persons with BD-I highlights the need to screen early for learning disability, including a primary intellectual disability or dysfunction related to mental illness such as depression in order to guide clinically appropriate treatment (Wilkinson et al. 2007). Assessments and treatments targeting impairments in executive functioning, attention, verbal and working memory, and visuospatial functioning found consistently in BD patients are further encouraged by their identified associations with violence, manic symptoms and impulse dyscontrol (Huxley and Baldessarini 2007; Wingo et al. 2009). Specifically, intellectual disability, ranging from mild to severe, has been associated with increasing risk of violence-against-others and against-self (Tsiouris et al. 2011). Impairment in executive functioning, in particular, has been found to predict later diagnosis of BD among adolescents (Meyer et al. 2004).

The significant association found here among physically aggressive behaviors, suicide attempt, learning disability, and alcohol abuse may also be interpreted in light of recent conceptualizations of candidate endophenotypes of vulnerability to suicide, including impulsive aggression as well as disadvantageous decision making and deficits in risk assessment, with possibly related disturbances in prefrontal cortical functioning (Mann and Currier 2010; Courtet and Guillaume 2011). Also, a staging perspective on the psychopathology of BD-I might encourage speculation that an early learning disability, with its impact on comprehension, judgment and communication, may pave the way to disruptive social interactions, altered interpersonal skills, isolation, struggle for acceptance, and subjective experience of not fitting-in socially, particularly during adolescence, and ultimately undermine the quality of social connectedness that is proposed as a major protective factor from suicide (Salvatore et al. 2014). In addition, early distortions of social competence and constant strife arising from rejection by peers among youths with learning disabilities might also lead to substance misuse, in particular excessive alcohol consumption, to cope with social anxiety and heightened stress sensitivity, with further susceptibility to depression, dysphoria, anger and impulsivity as well as suicidal risk and violence. More research is needed into the psychological wounds (Siegel 2016) suffered by those with intellectual disabilities.

However, despite significant associations with aggressive acts at intake, the probability, or positive predictive value, of the four significant factors was small, in part reflecting the relatively low prevalence of violent behavior (10.6%). Associations of specific factors were: 5/19 (26.3%) for the presence of a learning disability, 7/30 (23.3%) for a suicide attempt, 19/133 (14.3%) for co-occurring alcohol use disorder, and 15/134 (11.2%) intake manic episode polarity. Therefore, the current findings have limited ability to predict future aggressive acts among bipolar disorder patients.

### Limitations

This study is limited by the moderate number of subjects, the low frequency of some factors, and the absence of uniform clinical assessment of certain factors, including learning disability, tested for association with aggressive behavior. Generalizability of findings to other BD-I patients may be limited by sampling from hospitalized subjects with psychotic features. Prevalence of serious aggression was identified by clinical assessment at hospital admission and by chart review, which may over- or under-evaluate the risk.

## Conclusions

Among 216 subjects with a stable diagnosis of BD-I with psychotic features at 2 years, aggressive behaviors were found in 23 (10.6%) at first-lifetime hospitalization—a minority, but a substantial risk. Such patients also had nearly five-fold greater odds of recent suicide attempt. Other factors associated with aggression included alcohol abuse, learning disability, and initial presentation in mania rather than other forms of illness. Some of these factors are potentially modifiable, including with early educational and therapeutic efforts to support social-emotional well-being and skills required for personal and vocational functioning. We propose that such efforts may contribute to reducing risk of violent behavior and injuries or deaths associated with a minority patients with BD-I.

## Abbreviations

BD: bipolar disorder
BD-I: bipolar I disorder
SD: standard deviation
CI: confidence intervals
OR: odds ratios
ID: intellectual disability
SCID-P: structured clinical interview for DSM
DSM-IV-TR: Diagnostic and Statistical Manual-IV-Treatment Version
BPRS-E: Expanded Brief Psychiatric Rating Scale
PS: Dr. Paola Salvatore
